# Adherence to Self-Care among Patients with Hypertension in Ethiopia: A Systematic Review and Meta-Analysis

**DOI:** 10.1155/2022/5962571

**Published:** 2022-07-16

**Authors:** Afework Edmealem, Sewunet Ademe, Sisay Gedamu

**Affiliations:** Department of Nursing, School of Nursing and Midwifery, Wollo University, Dessie, Ethiopia

## Abstract

**Background:**

Self-care adherence for hypertensive patients is a cornerstone for the control of it and prevention of its complications. However, there are inconsistent findings for self-care adherence of hypertensive patients in Ethiopia. Thus, this systematic review and meta-analysis was done to determine the pooled estimate self-care adherence.

**Methods:**

This systematic review and meta-analysis was reported based on the Preferred Reporting Items for Systematic Review and Meta-Analysis guideline. An intensive search of online databases such as PubMed (MEDLINE), CINHAL, Google Scholar, and advanced Google search was made to access both published and unpublished articles that report self-care adherence among hypertensive patients in Ethiopia. The pooled estimate was done with STATA version 11 metan commands in a 95% confidence interval. The presence of heterogeneity and publication bias were detected by I^2^ and Egger's test, respectively. A random-effect model was obtained, and subgroup analysis was done for the management of heterogeneity.

**Result:**

A total of 24 articles with a total of 7224 participants were included in the final systematic review and meta-analysis. The pooled estimate of overall self-care adherence among hypertensive patients was 36.98% (95% CI: 27.13–46.83). In subgroup analysis, the highest overall self-care adherence was observed in the Amhara region at 53% (95% CI: 46.54, 59.47). The pooled estimate of self-care dimensions such as medication adherence, low-salt diet, physical activity, smoking abstinence, alcohol abstinence, and weight management was 62.71%, 65.96%, 47.28%, 92.53%, 67.59%, and 52.54%, respectively.

**Conclusion:**

The pooled estimate of good self-care adherence among hypertensive patients was low. From the dimensions of self-care, the lowest level was in physical activity and the highest level was in smoking abstinence. Comparing all regions, the lowest level of overall self-care adherence was observed in Addis Ababa, Tigray region, and South Nations and Nationalities of Ethiopia. Screening of adherence to self-care and health education should be provided to every patient during every visit.

## 1. Background

Globally, the prevalence of hypertension increased from 594 million in 1975 to 1.13 billion in 2015 with a high increase in low- and middle-income countries [1]. In Ethiopia, the prevalence of hypertension is 30% [2] and from these, 48% of them had uncontrolled hypertension [3]. Non-communicable diseases are the common cause of death in Ethiopia. Of the total deaths, 16% of them are due to cardiovascular diseases such as uncontrolled hypertension [4].

Uncontrolled hypertension leads to heart attack, stroke, heart failure, and other serious illnesses [5].The impact of uncontrolled hypertension increased in sub-Saharan African countries over the past few decades [6]. To prevent the crisis of uncontrolled hypertension, patients need to adhere to effective lifestyle modifications [7].

The World Health Organization (WHO) defines self-care as the ability of individuals, families, and communities to promote and maintain health, prevent illness, and cope with illness and disability with or without the assistance of a health professional [8]. Self-monitoring of blood pressure lowers blood pressure in patients with hypertension more than the usual care [9]. Controlling blood pressure at home has a potential advantage to prevent complications [10]. Despite this fact, poor adherence to the recommended healthy lifestyle is one of the most common reasons for uncontrolled hypertension, serious complications, and high health care expenditure [11].

Self-care adherence for hypertension includes medication adherence, adoption of a low-salt diet, regular physical activity for 30 minutes on most days, loss or maintenance of weight, limiting alcohol intake, and ceasing tobacco use [12]. Adherence to these six self-care practices is important to control hypertension and prevent its devastating complications [13, 14]. Not only automated self-care support but telemonitoring of patients self-care behavior also reduces uncontrolled systolic hypertension and improves control of hypertension [15].

Adherence to self-care decreases health care expenditure and morbidity and mortality from potentially preventable complications such as stroke and heart disease. It also improves patients' daily functions. This in turn increases activities of daily living and quality of life [6].

The level of self-care adherence among hypertensive patients in Ethiopia was reported by different studies at different time periods which ranges from 20.3% to 59.4% [11, 16–23]. However, these findings are inconsistent. The level of self-care adherence among hypertensive patients at the national level is not known. It is difficult to make policy, evaluate the treatment guideline, and design and plan interventions for patients with poor adherence to self-care without understanding the pooled evidence of self-care adherence. Thus, this systematic review and meta-analysis is done to determine the pooled estimate of good self-care among hypertensive patients at the national level.

## 2. Methods

### 2.1. Study Design and Setting

This study was conducted through a systematic review and meta-analysis of both published and unpublished articles conducted in Ethiopia. Ethiopia is one of the developing countries in the East part of Africa. It has 9 regions and 2 city administrations. The regions are Tigray, Afar, Amhara, Oromo, Ethiosomali, Benshangul-Gumuz, South Nations and Nationalities of People, Gambela, and Harar. The two city administrations are Addis Ababa and Dredawa [24]. A recent systematic review and meta-analysis showed that the prevalence of hypertension in Ethiopia is 19.6% [2].

### 2.2. Data Source and Search Strategy

Both published articles and grey literature that assessed self-care adherence of hypertensive patients in Ethiopia was used as sources of data. The finding of this systematic review and meta-analysis was carried out based on the Preferred Reporting Items for Systematic Review and Meta-Analysis (PRISMA) guideline [25]. The search strategy was developed using Population Intervention Comparison and Outcome (PICO) searching guide. For published articles, an intensive search of online data bases such as PubMed (MEDLINE), CINHAL, PsycINFO, Cochrane, Africa Journal of Online, Google Scholar, and advanced Google search was made. Grey literature such as agency reports, governmental articles, and academic thesis was intensively searched from the online library of governmental and academic institutions in Ethiopia such as Addis Ababa University. During the systematic search, the word ((((((“self care behavior”) OR “self care adherence”) OR “self care” OR “dietary practice” OR “weight management” OR “Physical activity” OR “medication adherence”) AND “hypertensive patients”) OR “patients with hypertension”) AND Ethiopia) was used. All articles in reference lists were searched to include additional studies and reports in the review and analysis. A systematic search of the literature was made from January 21^st^ till May 1^st^ 2020.

### 2.3. Eligibility Criteria

#### 2.3.1. Inclusion Criteria

In this systematic review and meta-analysis, all published and unpublished articles which are done in Ethiopia are included. All studies which reported either self-care adherence or low-salt diet or weight management or physical activity or medication adherence or alcohol and smoking abstinence among patients whose age is ≥ 18 years and with hypertension were included. All cross-sectional studies published in the English language and conducted among all stages of hypertensive patients and published from May 2000 until May 2020 were included.

#### 2.3.2. Exclusion Criteria

Studies that report self-care adherence among patients with pregnant women were excluded. In addition, studies that fail to report either self-care practice or dimensions of self-care practice such as weight management, physical activity, dietary management, smoking habit, alcohol drinking, and medication adherence were not included. Studies that are case reports, case series and studies that do not report primary data were also excluded from the study.

### 2.4. Measure of Outcomes

#### 2.4.1. Hypertension Self-Care Adherence

Good hypertension self-care adherence is described as adherence to all components of self-care for hypertensive patients: medication adherence, physical activity, low-salt diet, smoking abstinence, alcohol drinking abstinence, and weight management [26, 27].

#### 2.4.2. Medication Adherence

Patients who scored ≥3 in a 4-item medication adherence scale or ≥6 in an 8-item medication adherence scale were categorized as adherent to medication.

#### 2.4.3. Physical Activity

Patients who do physical activity at least 30 minutes per day for at least 5 days per week were adherent to physical activity.

#### 2.4.4. Low-Salt Diet

Participants who took salt-free diet while cooking and eating for at least 6 or more days out of 7 days were said to be adherent to a low-salt diet.

#### 2.4.5. Smoking Abstinence

Participants who did not smoke ever or stopped smoking were called smoking abstainers.

#### 2.4.6. Alcohol Abstinence

Participants who did not drink any alcohol in the last 7 days were called alcohol abstainers.

#### 2.4.7. Weight Management

Patients who maintain their BMI at 18.5 kg/m^2^–24.99 kg/m^2^ during the study period were categorized as adherent to weight management.

#### 2.4.8. Quality Assessment and Critical Appraisal

In this systematic review and meta-analysis, the quality of included studies was assessed by an 8-item critical appraisal tool adopted from JBI [28]. This is a tool used for the evaluation of prevalence studies. SG and SA assessed the methodological quality of eligible articles independently. Any disagreement among the authors was solved by discussion and consensus. All articles that scored above half of the score were included in the systematic review and meta-analysis.

#### 2.4.9. Data Extraction/Abstraction

All the authors extracted the data from the included literature in prepiloted format which is prepared in a Microsoft Excel spread sheet. From each article, the name of the first author, year of publication, region, sample size, self-care adherence, adherence to low-salt diet, weight management, smoking abstinence, alcohol abstinence, physical activity, and medication adherence were extracted. AE collected the extracted data from the two authors, and any disagreement among the authors was solved by discussion.

### 2.5. Statistical Analysis

The extracted data were exported from the Microsoft Excel spread sheet into the command window of STATA version 11. Before running the main meta-analysis, the presence of statistical heterogeneity within the included articles was assessed using I-squared statistic. Accordingly, heterogeneity was classified as low, moderate, or high when the I-squared values were 25, 50, and 75%, respectively. A value of zero indicates the presence of no heterogeneity. In addition, the presence of publication bias was assessed with Egger's tests. After that, the pooled estimate was done by the STATA metan command. Because of the presence of heterogeneity within articles, the pooled estimate of self-care practice, adherence to a low-salt diet, weight management, physical activity, smoking abstinence, alcohol abstinence, and medication adherence among hypertensive patients was done according to the Der Simonian-Laird random-effects model. Subgroup analysis of included studies by region was also done to manage heterogeneity. Additional advanced statistical analyses such as meta-regression to identify the potential sources of heterogeneity and sensitivity analysis to investigate the influence of a single study on the overall pooled estimate were performed. The findings of this study were presented using tables and forest plots with 95% confidence intervals (CIs).

### 2.6. Heterogeneity Test and Publication Bias

Heterogeneity within included articles was assessed statistically by using I-squared statistic. Accordingly, heterogeneity was classified as low, moderate, or high when the value of I-squared was 25 and below, 50, and 75% and above, respectively. The presence of publication bias was statistically assessed by Egger's weighted regression method. *P* value < 0.05 was considered as presence of significant publication bias.

## 3. Results

### 3.1. Identification of Studies

**Figure 1:**
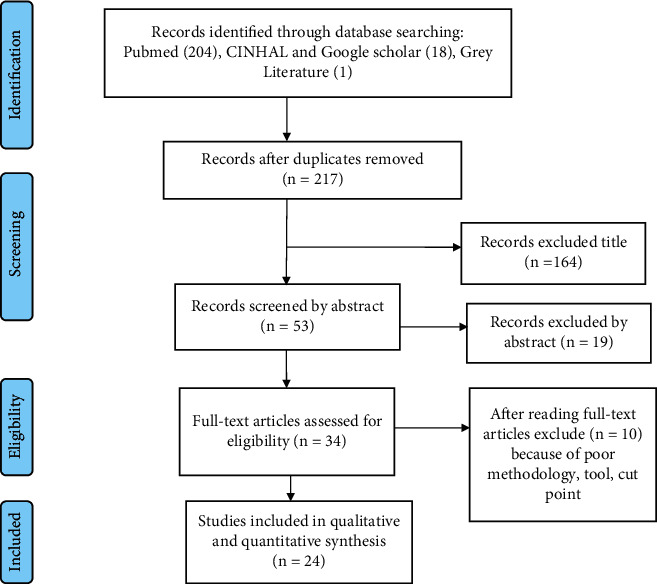
PRISMA flow diagram that shows study selection for meta-analysis of self-care adherence for hypertensive patients.

A total of 223 articles were retrieved from PubMed, CINAHL, Google Scholar, and Google through advanced searching. Among the total articles, 5 of them were added through reference tracing. After removing 6 duplicated articles, 217 articles were screened by their title and abstract. One hundred eighty-three articles were removed by reviewing their title and abstract, and the rest 34 articles were screened by full-text review. After that, 24 articles that pass the quality assessment and eligibility criteria were included in the final systematic review and meta-analysis (Figure 1).

### 3.2. Description of Included Studies

A total of 24 articles with 7224 study participants were included in the final systematic review and meta-analysis. Among these, 9 and 8 articles were done in the Oromia region [11, 22, 23, 27, 29–33] and the Amhara region [16, 19, 20, 34–38], respectively. The other three articles were conducted in the Tigray region [26, 27, 39], three articles were in Addis Ababa [18, 40, 41], and one article was conducted in South Nations and Nationalities of People [17]. The largest sample size was 616 which was obtained from a study conducted in Addis Ababa in 2016 [41]. Of the total articles, only 9 of them [11, 16–18, 20, 22, 23, 26, 38] reported the overall self-care adherence of hypertensive patients (Table 1).

### 3.3. Adherence to Self-Care among Hypertensive Patients

**Figure 2:**
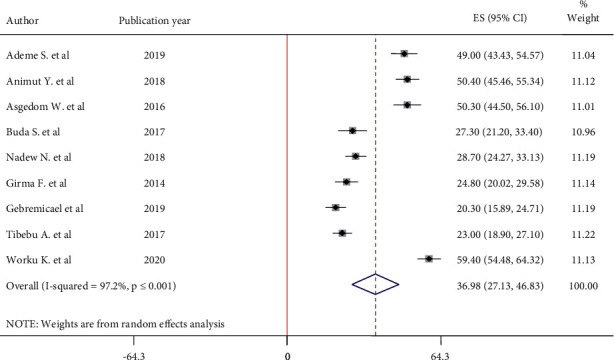
Forest plot depicting the pooled prevalence of self-care adherence among hypertensive patients in Ethiopia, 2020.

Of the total of 24 articles, only nine articles reported overall self-care adherence and were included in the meta-analysis. Before performing the main meta-analysis, the presence heterogeneity was detected by STATA version 11. According to the result, there is a presence of high heterogeneity between the studies. As a result, the main meta-analysis was performed by using a random-effect model, and subgroup analysis, meta-regression, and sensitivity analysis were done for the management of heterogeneity. The pooled estimate of self-care adherence among hypertensive patients from 9 studies was 36.98% (95% CI: 27.13, 46.83) (Figure 2). Heterogeneity between studies was high (I^2^ = 97.2%, *P* ≤ 0.001). The meta-regression test was performed by using sample size and publication year as covariates; however, both sample size and publication year are not the sources of heterogeneity (Table 2). The presence of publication bias was performed by Egger's test. According to the result, there is no evidence of publication bias (*P*=0.157). The result of sensitivity analysis using the random-effect model indicates that there is no single study that affects the pooled estimate self-care adherence among hypertensive patients.

### 3.4. Subgroup Analysis for Overall Self-Care Adherence

**Figure 3:**
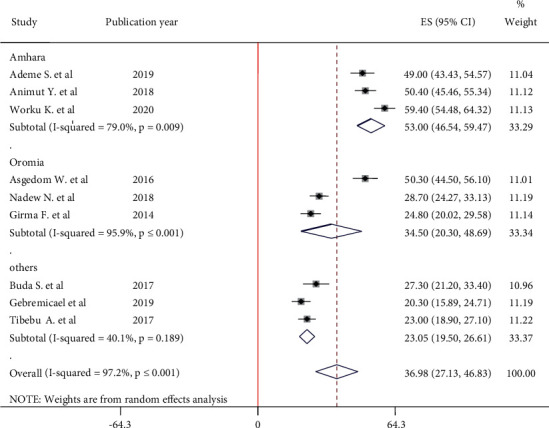
Subgroup analysis by region for self-care adherence of hypertensive patients in Ethiopia, 2020.

Subgroup analysis is done by study area (region) for the management of high heterogeneity. As it is shown in Figure 3, the highest prevalence of good self-care adherence was observed in the Amhara region at 53% (95% CI: 46.54, 59.47) followed by the Oromia region at 34.50% (95% CI: 20.30, 48.69) and the lowest prevalence of good self-care adherence was observed in Tigray Region, Addis Ababa, and SNNP at 23.05% (95% CI: 19.50, 26.61) (Figure 3).

### 3.5. Hypertension Self-Care Adherence by Dimensions

#### 3.5.1. Medication Adherence

**Figure 4:**
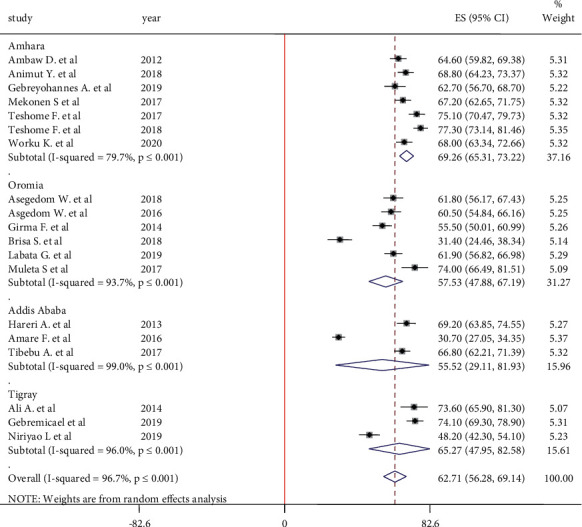
Subgroup analysis for medication adherence among hypertensive patients in Ethiopia.

In this meta-analysis, a total of 19 studies were included. The pooled estimate of medication adherence among hypertensive patients with a random-effect model was 62.71% (95% CI: 56.72, 69.14). Because of the presence of high heterogeneity (I^2^ = 96.7%, *P* ≤ 0.001), subgroup analysis by study region was done. According to the finding, the highest prevalence of medication adherence was observed in Amhara region at 69.26% (95 CI: 65.31, 73.22), followed by medication in Tigray region at 65.27% (95% CI: 47.95, 82.58) (Figure 4).

#### 3.5.2. Adherence to Low-Salt Diet

**Figure 5:**
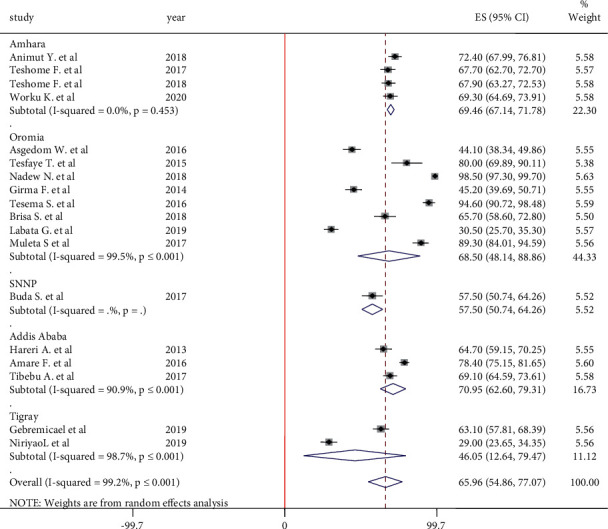
Forest plot on adherence to low-salt diet among hypertensive patients in Ethiopia.

A total of 19 articles were included to pool estimate adherence to the low-salt diet. According to the finding, the pooled estimate of adherence to a low-salt diet with a random-effect model was 65.96% (95% CI: 54.86, 77.07). Because of the presence of high heterogeneity (I^2^ = 99.2%, *P* ≤ 0.001) within the studies, subgroup analysis was done by region. According to the result, the highest adherence to a low-salt diet was observed in Addis Ababa at 70.95% (95% CI: 62.60, 79.31), followed by the Amhara region at 69.46% (95% CI: 67.14, 71.78). Hypertensive patients in the Tigray region had the lowest adherence to a low-salt diet at 46.05% (95% CI: 12.64, 79.47) compared to hypertensive patients in other regions of the country (Figure 5).

#### 3.5.3. Adherence to Physical Activity

**Figure 6:**
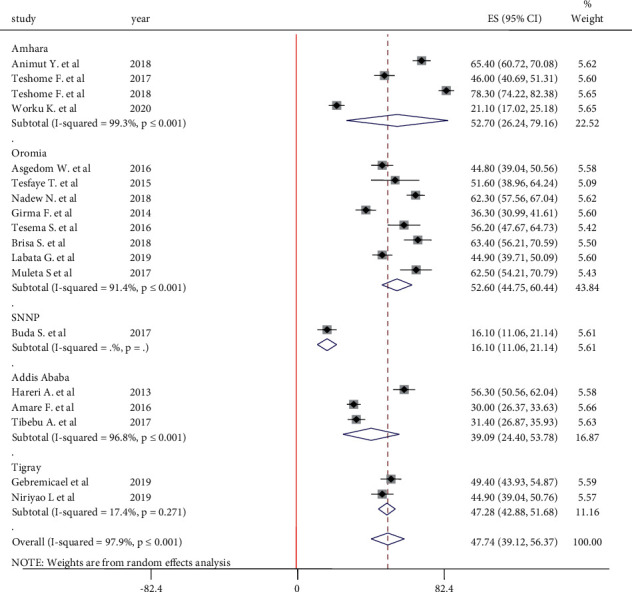
Forest plot on adherence to physical activity among hypertensive patients in Ethiopia.

A total of 18 articles reported adherence of hypertensive patients to physical activity. The highest level of adherence to physical activity was reported by Tibebu et al. [18] in the Amhara region. In this meta-analysis, the pooled estimate of adherence to physical activity in the random-effect model 47.74% (95% CI: 39.14, 56.37). Subgroup analysis showed that 47.28% (95% CI: 42.88, 51.68) of hypertensive patients in the Tigray region were adherent to physical activity. Adherence to physical activity among hypertensive patients in the Amhara region and the Oromia region is nearly similar (Figure 6).

#### 3.5.4. Adherence to Smoking Abstinence

**Figure 7:**
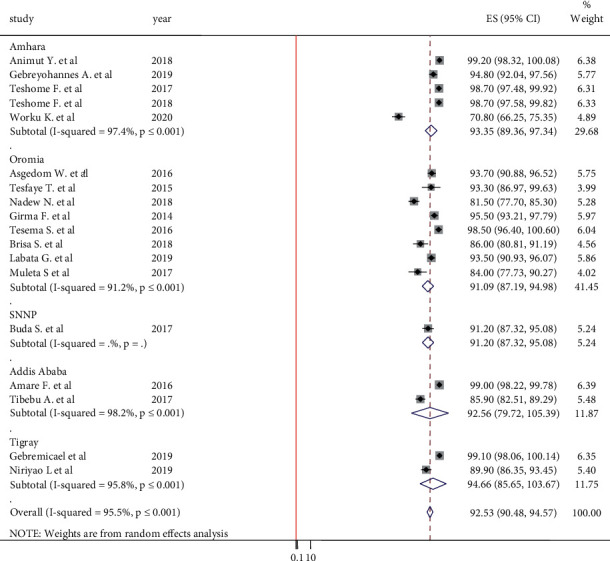
Forest plot on adherence to smoking abstinence among hypertensive patients.

The lowest and highest adherences to smoking abstinence were reported by Worku K et al. [38] and Animut Y et al. [20] in Amhara region, respectively. A total of 18 articles were included to determine the pooled estimate of adherence to abstinence in hypertensive patients. The pooled estimate of adherence to smoking abstinence with the random-effect model was 92.53% (95% CI: 90.48, 94.57). This indicates that close to 7% of hypertensive patients were smoking. According to the subgroup analysis, the lowest adherence to smoking abstinence was observed in the Oromia region of 91.09% (95% CI: 87.19, 94.98) (Figure 7).

#### 3.5.5. Adherence to Alcohol Abstinence

**Figure 8:**
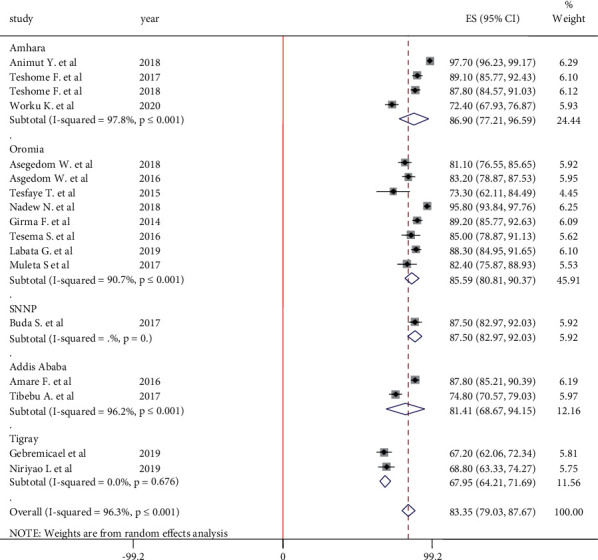
Forest plot on adherence to alcohol abstinence among hypertensive patients in Ethiopia.

The pooled estimate of adherence to alcohol abstinence was done from the report of 17 articles. However, because of high heterogeneity (I^2^ = 96.3, *P*=0.001) between studies, the overall pooled estimate was done using the random-effect model and subgroup analysis was done by region. According to the result, the highest adherence to alcohol abstinence among hypertensive patients was observed in the Amhara region at 89.6% (95% CI: 77.21, 96.59) and the lowest was in the Tigray region at 67.59% (95% CI: 64.21, 71.69). In the Tigray region, only 64.21% of hypertensive patients abstained from alcohol drinking (Figure 8).

#### 3.5.6. Weight Management

**Figure 9:**
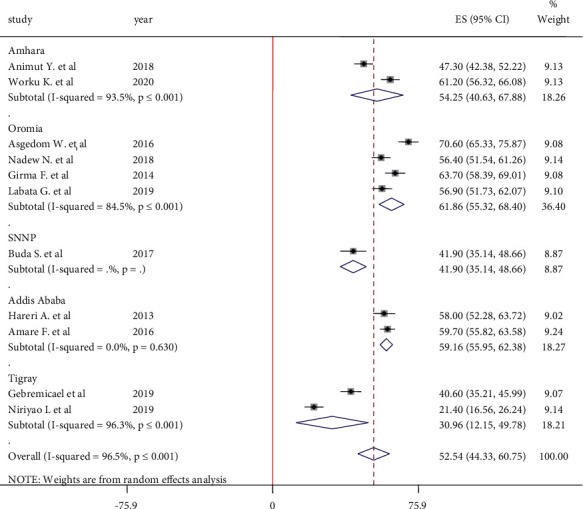
Forest plot on adherence to weight management among hypertensive patients in Ethiopia.

The pooled estimate of weight management in this meta-analysis was done from the report of 11 articles. Because of high heterogeneity (I^2^ = 96.5%, *P* ≤ 0.001), both the main meta-analysis and subgroup analysis were done by using the random-effect model. The pooled estimate of weight management was 52.54% (95% CI: 44.33, 60.75). Hypertensive patients in the Oromia region had the highest adherence to weight management at 61.86% (95% CI: 55.32, 68.40) (Figure 9).

## 4. Discussion

Self-care adherence for hypertensive patients is a cornerstone for the control of hypertension and its complication. This systematic review and meta-analysis was done to determine the pooled estimate of self-care adherence among hypertensive patients with its six dimensions (medication adherence, low-salt diet, physical activity, smoking abstinence, alcohol abstinence, and weight management) from the reports of 24 articles.

In this systematic review and meta-analysis, overall self-care adherence of hypertensive patients was determined from 9 articles with a total of 3018 study participants. According to the result, the pooled estimate of overall good self-care adherence among hypertensive patients was 36.98% (95% CI: 27.13, 46.83). This indicates that hypertensive patients who had good self-care adherence to control their hypertension were low. So, the majority of patients with hypertension were at high risk for uncontrolled hypertension and its devastating complications such as stroke and heart diseases. This finding is in line with a multicenter study conducted in China (32.9%) [42]. However, the finding of this systematic review and meta-analysis is lower than the finding of the survey conducted in Southwest Nigeria (61.2%) [43] and a nationwide population-based study in Korea at 72.5% [44] and Saudi Arabia at 74.4% [45]. The possible justification for this discrepancy could be the difference in study design, sample size, and sociodemographic variation.

Adherence to medication for hypertensive patients is the main way to control their blood pressure. In this meta-analysis, the pooled estimate of medication adherence among hypertensive patients is 62.71% (95% CI: 56.72, 69.14). This indicates that only 62.71% of hypertensive patients were taking all their medication as prescribed. This finding is lower than the previous studies which are conducted in Saudi Arabia (83.7%) [45] and Uganda (79.5%) [46]. However, the finding of this study was higher than a study conducted in Southern Iran (36.1%) [47]. The possible reason for this difference could be the difference in the healthcare system. The difference in health perception and management between the study participants could be another reason for the discrepancy. In addition, study participants in the study conducted in Southern Iran were in the age group of 30–90 years which did not include participants less than 30 years unlike this study. The use of different tools to assess medication adherence between the studies might be the other difference.

The other dimension of self-care for hypertension is adherence to low-salt diet. This study reported that the pooled estimate of adherence the low-salt diet was 65.96% (95% CI: 54.86, 77.07). This finding is in line with the study conducted in Uganda (75.6%) [46] and Kenya (63%) [48]. However, the finding of this meta-analysis is lower than a study conducted in Saudi Arabia (79.3%) [45]. The reason for this could the difference in tools and dietary habits of the population.

Hypertensive patients need to do regular physical activity for at least 30 minutes on at least 5 days of a week [49]. Physical activity prevents left ventricular hypertrophy of the hypertensive heart among hypertensive patients [50]. The pooled estimate of adherence to the recommended physical activity was 47.74% (95% CI: 39.14, 56.37). More than half of hypertensive patients were not adherent to the recommended physical activity. The finding of this meta-analysis is in line with the finding of study conducted in Saudi Arabia (57.3%) [45] and a nationwide population-based study in Korea at (44.08%) [44]. But, this is lower than a study conducted in Uganda (63.1%) [46] and Kenya (67%) [48]. The possible justification for this discrepancy might be the difference in the study population. A study conducted in Uganda took study participants from a rural area so that these individuals will have better engagement in physical activity during daily activities such as farming.

Smoking cigarette increases the constriction of blood vessels and heart activity. Thus, hypertensive patients should stop smoking for the control of their blood pressure [51]. In this meta-analysis, the pooled estimate of adherence to smoking abstinence was 92.53% (95% CI: 90.48, 94.57). This is in line with a study conducted in Uganda at 98.7% [46]. This finding is higher than the study conducted in China (72.6%) [42], Saudi Arabia (31.2%) [45], and nationwide population-based study in Korea (87.39%) [44]. The possible reason for this difference could be socioeconomic difference. On the other hand, the pooled estimate of adherence to alcohol abstinence was 83.35 (95% CI: 79.03, 87.67). This finding is higher than study conducted in China (77.5%) [42] and lower than study conducted in Uganda (90.1%) [46].

Weight loss is one of the most effective lifestyle changes for controlling blood pressure. Losing one kilogram weight decreases one millimeter mercury (mmHg) [52]. In this meta-analysis, the pooled estimate of adherence to weight management was 52.54% (95% CI: 44.33, 60.75). Only 52.54% hypertensive patients were maintaining their normal weight. This finding is in line with a study conducted in Saudi Arabia (59.9%) [45] and Korea (51.28%) [44]. However, this is higher than study conducted in China (34.8%) [42] and Southern Iran (39.2%) [47] and lower than study conducted in Uganda (75.6%) [46] and Kenya (90.5%) [48].

### 4.1. Limitation

This systematic review and meta-analysis has its own limitations. The first limitation is the included articles used different tools for the measurement the outcomes. All articles report the prevalence of self-care adherence based on patients' self-report. Thus, this could be another limitation for this study.

## 5. Conclusion

In this systematic review and meta-analysis, the pooled estimate of good adherence to self-care among hypertensive patients was low. The lowest adherence to self-care was observed in physical activity and the highest was in smoking abstinence. The lowest adherence was observed in Addis Ababa, Tigray region, and SNNP of Ethiopia. Health care providers should encourage patients to adhere to self-care activities. Screening of adherence to self-care in every visit for every patient should be done. Researchers need to identify factors that affect the self-care adherence of hypertensive patients.

## Figures and Tables

**Table 1:** Characteristics of included studies in the final systematic review and meta-analysis, 2020.

Author	Publication year	Region	Sample size	Reported outcome percentage						
				Overall self-care	Medication adherence	Low-salt diet	Physical activity	Smoking	Alcohol	Weight management
Ademe S et al.	2019	Amhara	309	49						
Ambaw D et al.	2012	Amhara	384		64.6					
Animut Y et al.	2018	Amhara	395	50.4	68.8	72.4	65.4	99.2	97.7	47.3
Asegedom W et al.	2018	Oromia	286		61.8				81.1	
Asegedom W et al.	2016	Oromia	286	50.3	60.5	44.1	44.8	93.7	83.2	70.6
Buda et al.	2017	SNNP	205	27.3		57.5	16.1	91.2	87.5	41.9
Hareri A et al.	2013	Addis Ababa	286		69.2	64.7	56.3			58
Ali A et al.	2014	Tigray	126		73.6					
Tesfaye T et al.	2015	Oromia	60			80	51.6	93.3	73.3	
Nadew N et al.	2018	Oromia	401	28.7		98.5	62.3	81.5	95.8	56.4
Girma F et al.	2014	Oromia	314	24.8	55.5	45.2	36.3	95.5	89.2	63.7
Amare F et al.	2016	Addis Ababa	616		30.7	78.4	30	99	87.8	59.7
Tesema S et al.	2016	Oromia	130			94.6	56.2	98.5	85	
Berisa S et al.	2018	Oromia	172		31.4	65.7	63.4	86		
Geberemica el al.	2019	Tigray	320	20.3	74.1	63.1	49.4	99.1	67.2	40.6
Gebereyohannes et al.	2019	Amhara	249		62.7			94.8		
Labeta G et al.	2019	Oromia	352		61.9	30.5	44.9	93.5	88.3	56.9
Mekonnen S.et al.	2017	Amhara	409		67.2					
Muleta S et al.	2017	Oromia	131		74	89.3	62.5	84	82.4	
Nariyo L et al.	2019	Tigray	276		48.2	29	44.9	89.9	68.8	21.4
Teshome F et al.	2017	Amhara	337		75.1	67.7	46	98.7	89.1	
Teshome F et al.	2018	Amhara	392		77.3	67.9	78.3	98.7	87.8	
Tibebu A et al.	2017	Addis Ababa	404	23	66.8	69.1	31.4	85.9	74.8	
Worku K et al.	2020	Amhara	384	59.4	68	69.3	21.1	70.8	72.4	61.2

**Table 2:** Meta-regression analysis for heterogeneity of self-care adherence among hypertensive patients in Ethiopia, 2020.

Overall self-care adherence	Coefficients	Std. error	*P* value
Year	3.216225	3.254573	0.361
Sample size	0.0059339	0.0883559	0.949

## Data Availability

All the data are included in this document but if any, the corresponding author can provide upon reasonable request.

## Abbreviations

JBI: Joanna Briggs Institute
PRISMA: Preferred Reporting Items for Systematic Review and Meta-Analysis
PICO: Population Intervention Comparison Outcome
SNNP: South Nations and Nationalities of People
WHO: World Health Organization.
